# Matcha Improves Metabolic Imbalance-Induced Cognitive Dysfunction

**DOI:** 10.1155/2020/8882763

**Published:** 2020-11-28

**Authors:** Jong Min Kim, Uk Lee, Jin Yong Kang, Seon Kyeong Park, Jong Cheol Kim, Ho Jin Heo

**Affiliations:** ^1^Division of Applied Life Science (BK21 Plus), Institute of Agriculture and Life Science, Gyeongsang National University, Jinju 52828, Republic of Korea; ^2^Division of Special Purpose Tree, National Institute of Forest Science, Suwon 16631, Republic of Korea; ^3^Institute of Hadong Green Tea, Hadong 52304, Republic of Korea

## Abstract

This study was conducted to assess the protective effect of extract of match (EM) on high-fat diet- (HFD-) induced cognitive deficits in male C57BL/6 mice. It was found that EM improved glucose tolerance status by measuring OGTT and IPGTT with HFD-induced mice. EM protected behavioral and memory dysfunction in Y-maze, passive avoidance, and Morris water maze tests. Consumption of EM reduced fat mass, dyslipidemia, and inflammation in adipose tissue. Also, EM ameliorated hepatic and cerebral antioxidant systems. EM improved the cerebral cholinergic system by regulating ACh contents and expression of AChE and ChAT. Also, EM restored mitochondrial function in liver and brain tissue. EM attenuated hepatic inflammatory effect, lipid synthesis, and cholesterol metabolism by regulating the protein expression of TNF-*α*, TNFR1, *p*-IRS-1, *p*-JNK, IL-1*β*, iNOS, COX-2, HMGCR, PPAR*γ*, and FAS. Finally, EM regulated cognitive function and neuroinflammation in the whole brain, hippocampus, and cerebral cortex by regulating the protein expression of *p*-JNK, *p*-Akt, *p*-tau, A*β*, BDNF, IDE, COX-2, and IL-1*β*. These findings suggest that EM might be a potential source of functional food to improve metabolic disorder-associated cognitive dysfunction.

## 1. Introduction

Diabetes is one of the metabolic diseases caused by the consumption of a high-fat diet (HFD) and causes metabolic disorders such as dyslipidemia, insulin resistance, hepatic steatosis, and nonalcoholic fatty liver disease [1]. Long-term intake of HFD is one of the risk factors associated with diabetes and reduces the antioxidant system of the brain and liver [2]. In particular, the liver plays an important role in diabetes-related insulin resistance and hepatic glucose production by regulating gluconeogenesis and glycogenolysis by activating immune cells secreting inflammatory cytokines such as tumor necrosis factor-alpha (TNF-*α*) and interleukin 1 beta (IL-1*β*) [3, 4]. It has been reported that these inflammatory cytokines interfere with insulin signaling molecules such as insulin receptor substrate-1 (IRS-1) to phosphorylate serine/threonine kinases [5]. In addition, inflammatory cytokines produced in the liver move through the blood to the whole body and affect brain tissue [6].

Brain tissue is known to be a tissue that is susceptible to damage due to the inflammatory reaction caused by HFD [7]. Cognitive dysfunction induced by inflammation is associated with reactive oxygen species (ROS) production and mitochondrial damage in brain tissue, and impaired mitochondrial dysfunction continuously causes insulin resistance in cerebral neuronal cells [4, 8]. In particular, insulin resistance increases the problems associated with diabetic cognitive dysfunction, which leads to diseases such as Alzheimer's disease (AD) [9].

Matcha consumed worldwide as a processed powdered green tea (*Camellia sinensis*) is known to contain various phenolic compounds and bioactive substances such as tannins and catechins [10]. In particular, matcha has been reported to contain large amounts of catechins such as (−)-epicatechin, (−)-epigallocatechin, (−)-epicatechin-3-gallate, and (−)-epigallocatechin-3-gallate (EGCG) compared to other processed green tea such as hojicha, oolong tea, and black tea [11]. These catechins have various bioactive effects such as renal and neuroprotective effects, liver injury inhibitory effect, and antidiabetic effect derived from the structural features of flavan-3-ol [12–14]. As various studies about physiological activity have been reported, studies on using matcha as a healthy functional food are continuously increasing [10, 15].

However, there are few studies related to the protective effect of matcha against cognitive impairment caused by hepatic and cerebral insulin resistance. Therefore, by confirming the regulatory effect of hepatic inflammation on brain cognitive dysfunction, the improvement effect of matcha was studied in HFD-induced diabetic disorder mice.

## 2. Materials and Methods

### 2.1. Chemicals

Hydroxylamine·hydrochloride, phenylmethane sulfonylfluoride, thiobarbituric acid (TBA), metaphosphoric acid, o-phthaldialdehyde, bovine serum albumin (BSA), HEPES, digitonin, 2′,7′-dichlorofluorescein diacetate (DCF-DA), tetrachloro-1,1,3,3-tetraethylbenzimidazolylcarbo-cyanine iodide (JC-1), polyvinylidene difluoride, iron (III) chloride hexahydrate, protease inhibitor (PI), and polyvinylidene difluoride (PVDF) membrane were purchased from Millipore (Billerica, MA, USA). Superoxide dismutase (SOD) determination kit was purchased from Dojindo Molecular Technologies (Kumamoto, Japan). Primary antibody information is presented in Table S1. Secondary antibodies were purchased from Cell Signaling Technology (Danvers, MA, USA).

### 2.2. Sample Preparation

Matcha used in this experiment was provided by the Institute of Hadong Green Tea (Hadong, Korea). Samples are cultivated for 20-21 days to block the light for improvement of palatability. To minimize green color loss, samples were treated with superheated steam more than 250°C within 1 min. Then, steamed green tea was rapidly cooled by a cooler (900 K-1; Kawasaki Tea Machinery, Kakegawa, Japan). The cooled green tea was dried to a moisture content of less than 10% using a dryer (Kawasaki Tea Machinery) and powdered using a bead mill consist of millstone (Kawasaki Tea Machinery) and automatic mill stone (Kawasaki Tea Machinery). The rotational speed of the rotating upper millstone was 50 to 55 rpm, and particle size was crushed to be 13 to 14 *μ*m to produce matcha. To investigate the physiological activity of matcha, samples lyophilized using a freeze drier (Operon, Gimpo, Korea) at -80°C were extracted with 50-fold distilled water at 40°C for 2 h. The extracted sample was evaporated using a vacuum rotary evaporator (N-N series, Eyela Co., Tokyo, Japan). The concentrated sample was reevaporated at 40°C and lyophilized. The ultimately progressed extract of matcha (EM) was kept at -20°C until use.

### 2.3. Animals and In Vivo Experimental Design

Male 4-week-old C57BL/6 mice were purchased from Samtako (Osan, Korea). All animal experimental processes were approved at the Institutional Animal Care and Use Committee of Gyeongsang National University (certificate: GNU-161116-M0065) and were conducted according to the Policy of the Ethical Committee of Ministry of Health and Welfare (Republic of Korea). These mice were controlled in standard laboratory conditions with a 12 h light/dark cycle and 55% humidity at room temperature with free access to food and water. The mice were divided into 4 groups with each group. The control group was fed at normal diet (including protein (20 kcal%/g), carbohydrate (70 kcal%/g), and fat (10 kcal%/g), 3.85 kcal/g) for 14 weeks (NC). The HFD group and sample groups were fed at HFD (including protein (20 kcal%/g), carbohydrate (20 kcal%/g), and fat (60 kcal%/g), 5.24 kcal/g) for 14 weeks. The composition of experimental diets was presented in Table S2 [16]. The sample groups (EM 20 and EM 50) were intragastrically fed at EM (20 and 50 mg/kg of body weight, respectively).

### 2.4. Glucose Tolerance Test

Oral glucose tolerance test (OGTT) and intraperitoneal glucose tolerance test (IPGTT) were conducted in all groups. After, the mice were fasted for 4 h, and then D-glucose (2 g/kg of body weight) was orally in OGTT and intraperitoneally ingested in IPGTT. The concentration of glucose was measured using an Accu-Chek glucose meter (Roche Diagnostics, Basel, Switzerland).

### 2.5. Behavioral Test

#### 2.5.1. Y-Maze Test

Y-maze consists of the length (33 cm), height (15 cm), and width (10 cm), respectively. The mice were located at the end of the designated arm and allowed to freely move in the arms for 8 min [17]. The movement and path tracing were recorded using a video system (Smart 3.0, Panlab, Barcelona, Spain).

#### 2.5.2. Passive Avoidance Test

The chamber for the passive avoidance test was divided into an illuminated part and nonilluminated part that could give electrical stimulation. On the first day, the mice were located in an illuminated part. When four foot of the mice entered the nonilluminated part, a foot shock was applied at 0.5 mA for 3 s, and the first latency time was measured. In the test trial, the step-through latency to reenter the dark chamber was measured [18].

#### 2.5.3. Morris Water Maze (MWM) Test

MWM pool consists of a circular water pool (90 cm in diameter and 30 cm deep) and was randomly separated into four zones as N, S, E, and W. In the center of the W quadrant, a submerged platform below the water was located. The mice were allowed to swim freely, and their activities were recorded using a video system (Smart 3.0, Panlab). For the hidden trial test, each animal swam and escaped for four days. Lastly, a probe trial test was conducted without the platform, and retention time in the W zone was determined [19].

### 2.6. Serum Chemicals

Extraction of serum, liver, and brain was conducted as previously described [20]. The blood sample was collected at the postcaval vein and stored in a heparin tube. The supernatant centrifuged at 10,000 × *g* for 10 min at 4°C was immediately measured for serum biochemical assay. Glutamic oxaloacetic transaminase (GOT), glutamine pyruvic transaminase (GPT), lactate dehydrogenase (LDH), total cholesterol (TCHO), triglyceride (TG), and high-density lipoprotein cholesterol (HDLC) were measured by clinical chemistry analyzer (Fuji dri-chem4000i; Fuji film Co., Tokyo, Japan). Low-density lipoprotein cholesterol (LDLC) content and ratio of HDLC to TCHO (HTR) were calculated as follows [21].

(1) $$\begin{array}{c} \text{LDLC }\left(\frac{\text{mg}}{\text{dL}}\right)=\text{TCHO}-\left(\text{HDLC}+\frac{\text{TG}}{5}\right), \end{array}$$

(2) $$\begin{array}{c} \text{HTR }\left(\%\right)=\left(\frac{\text{HDLC}}{\text{TCHO}}\right)\times 100. \end{array}$$

### 2.7. Preparation of Tissue

Collected brain and liver were homogenized in a bullet blender (Next advance Inc., Averill Park, NY, USA) with 10-fold volumes of phosphate-buffered saline (PBS, pH 7.4) and phosphate buffer (pH 6.0) [22]. The protein concentration was measured using Bradford protein assay [23].

### 2.8. Antioxidant System

#### 2.8.1. Superoxide Dismutase (SOD) Contents

To confirm the antioxidant system, the homogenized tissue at PBS was spun down at 400 × *g* for 10 min at 4°C, and the pellets were measured for SOD analysis. These pellets in 5 volumes of 1 × cell extraction buffer (10% SOD buffer, 0.4% (*v*/*v*) Triton X-100, and 200 *μ*M phenylmethane sulfonylfluoride) were mixed at 10,000 × *g* for 10 min at 4°C. The SOD contents were measured using a commercial SOD kit (Dojindo Molecular Technologies).

#### 2.8.2. Reduced Glutathione (GSH) Contents

For reduced GSH analysis, tissues homogenized in phosphate buffer were centrifuged at 10,000 × *g* for 15 min at 4°C, and the supernatants were used for assay. This supernatant was reacted with 5% metaphosphoric acid and centrifuged 2,000 × *g*. The supernatant was reacted with 0.26 M tris-HCl (pH 7.8), 0.65 N NaOH, and 1 mg/mL of *o*-phthaldialdehyde at room temperature for 15 min. Then, the fluorescence intensity was measured using a fluorescence microplate reader (Infinite 200, Tecan Co., Männedorf, Switzerland) at a wavelength of 320 nm (excitation) and 420 nm (emission) [24].

#### 2.8.3. Malondialdehyde (MDA) Contents

To measure MDA contents, the homogenized tissues at PBS were centrifuged at 5,000 rpm for 10 min at 4°C. Supernatants were incubated with 1% phosphoric acid and 0.67% TBA in a 95°C water bath for 1 h. The reactants spun down at 600 × *g* for 10 min, and the supernatants were measured at 532 nm [25].

### 2.9. Cerebral Cholinergic System

#### 2.9.1. Acetylcholine (ACh) Contents

To confirm the cholinergic system, the brain homogenate at PBS was centrifuged at 14,000 × *g* for 30 min at 4°C, and the supernatants were used for the assay. To measure the ACh contents, supernatants and alkaline hydroxylamine reagent (2 M hydroxylamine·hydrochloride and 3.5 N sodium hydroxide (1 : 1)) were reacted at room temperature for 1 min, and then 0.5 N hydrochloride and 0.3 M iron (III) chloride hexahydrate were added. The absorbance was measured at a wavelength of 540 nm using a microplate reader (Epoch 2, BioTek Instruments Inc., Winooski, VT, USA) [26].

#### 2.9.2. Acetylcholinesterase (AChE) Activities

To measure the AChE activity, the samples were reacted with 50 mM sodium phosphate buffer (pH 7.4) at 37°C for 15 min, and then Ellman's reaction mixture was mixed at 37°C for 10 min. The absorbance was measured at a wavelength of 405 nm using a microplate reader (Epoch 2, BioTek Instruments Inc.) [27].

### 2.10. Mitochondrial Activity

#### 2.10.1. Mitochondrial Extraction

The tissues were homogenized with 10-fold volumes of mitochondrial isolation (MI) buffer (215 mM mannitol, 75 mM sucrose, 0.1% BSA, 20 mM HEPES sodium salt (pH 7.2)) with 1 mM EGTA. Homogenates were spun down at 1,300 × *g* for 10 min at 4°C, and the supernatants were centrifuged again at 13,000 × *g* for 10 min at 4°C. The mitochondrial pellets were mixed with MI buffer including 0.1% digitonin. After 5 min, mixtures were reacted with 2 ml MI buffer with 1 mM EGTA and centrifuged at 13,000 × *g* for 15 min at 4°C.

#### 2.10.2. Mitochondrial ROS Contents

Mitochondrial ROS production was measured using the isolated mitochondria with DCF-DA. The isolated mitochondria were added with KCl-based respiration buffer (125 mM potassium chloride, 2 mM potassium phosphate monobasic, 20 mM HEPES, 1 mM magnesium chloride, 500 *μ*M EGTA, 2.5 mM malate, and 5 mM pyruvate) and 25 *μ*M DCF-DA for 20 min. The DCF fluorescence was measured using a fluorescence microplate reader (Infinite 200, Tecan Co.) at 485 nm of excitation and 530 nm of emission [28].

#### 2.10.3. Mitochondrial Membrane Potential (MMP)

MMP was conducted using the isolated mitochondria with a 200 *μ*M JC-1. MI buffer with 5 mM pyruvate and 5 mM malate and isolated mitochondria was mixed with 1 *μ*M JC-1 in a black 96 well and reacted gently. The mixture was incubated at room temperature for 20 min at dark room, and then fluorescence was measured using a fluorescence microplate reader (Infinite 200, Tecan Co.) at excitation 530 nm and emission 590 nm [29].

### 2.11. Western Blot

The tissues were homogenized for 10 min in ice-cold extraction solution (GeneAll Biotechnology, Seoul, Korea) with 1% PI. The supernatants centrifuged at 13,000 × *g* for 10 min at 4°C were used for protein analysis. The protein runs through the SDS-PAGE gel and electrotransferred to the PVDF membrane. The membranes were reacted overnight in primary antibodies at 4°C and reacted with secondary antibodies for 1 h at room temperature. The immune complexes were detected using a western blot imager (iBright Imager, Thermo Fisher Scientific, Waltham, MA, USA), and the densities of protein expression were calculated using ImageJ software (National Institutes of Health, Bethesda, MD, USA) [30].

### 2.12. Statistical Analysis

All results presented as mean ± standard deviation. Statistically significant difference between each group was analyzed by one-way analysis and determined using Duncan's new multiple range test (*P* < 0.05) of SAS ver. 9.4 (SAS Institute Inc., Cary, NC, USA), and different small letters represent statistical differences.

## 3. Results

### 3.1. Body Weight Gain and Glucose Tolerance Test

#### 3.1.1. Body Weight Gain

**Figure 1:**
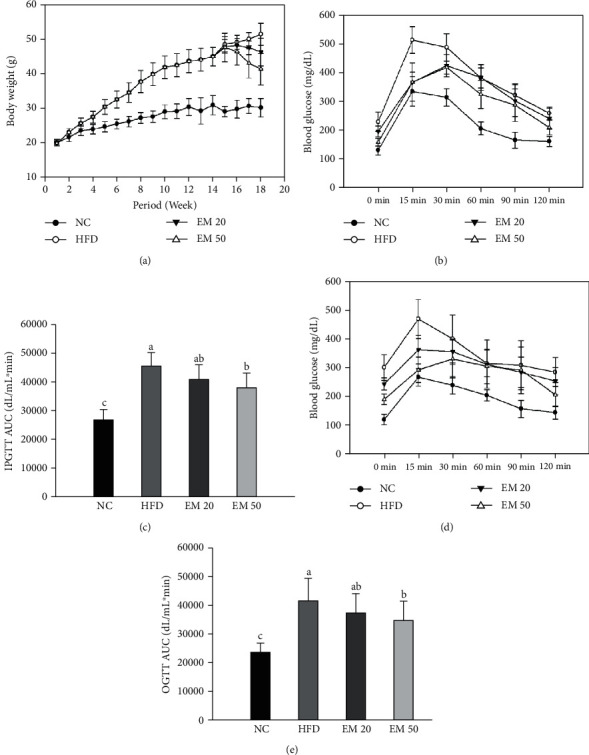
Effect of matcha on change of body weight (a), intraperitoneal glucose tolerance test (IPGTT) (b), AUC of IPGTT (c), oral glucose tolerance test (OGTT) (d), and AUC of OGTT (e). Results shown are mean ± SD (*n* = 7). Data were statistically considered at *P* < 0.05, and different small letters represent statistical differences.

The body weight changes of mice are presented in Figure 1(a). The body weight of the HFD group continuously increased from 15 to 18 weeks (48.40 to 51.30 g). However, the body weight of the EM 20 (47.70 to 46.20 g) and EM 50 (47.50 to 41.30 g) groups was statistically reduced more than the HFD group.

#### 3.1.2. IPGTT

IPGTT was conducted at 0, 15, 30, 60, 90, and 120 min (Figures 1(b) and 1(c)). Before the injection of glucose, the HFD group (228.57 mg/dL) showed a hyperglycemic state compared to the NC group (130.29 mg/dL), and the EM 20 and 50 groups measured 195.71 and 157.86 mg/dL, respectively. The NC and HFD groups showed the highest glucose level at 15 min (228.57 and 511.57 mg/dL, respectively), and the EM 20 and EM 50 groups showed the highest glucose levels at 30 min (426.29 and 417.00 mg/dL). The area under the curve (AUC) of the EM 20 and EM 50 groups (40563.22 and 37505.48 dL/mL∗min) was reduced more than that of the HFD group (45238.93 dL/mL∗min).

#### 3.1.3. OGTT

OGTT was conducted at 0, 15, 30, 60, 90, and 120 min (Figures 1(d) and 1(e)). Before the consumption of glucose, the EM 20 and 50 groups (243.83 and 191.00 mg/dL) showed a suppressed hyperglycemic state compared to the HFD group (301.00 mg/dL). The AUC of the EM 20 and EM 50 groups (36937.50 and 34264.50 dL/mL∗min) was reduced more than that of the HFD group (41223.50 dL/mL∗min).

### 3.2. Behavioral Tests

#### 3.2.1. Y-Maze Test

**Figure 2:**
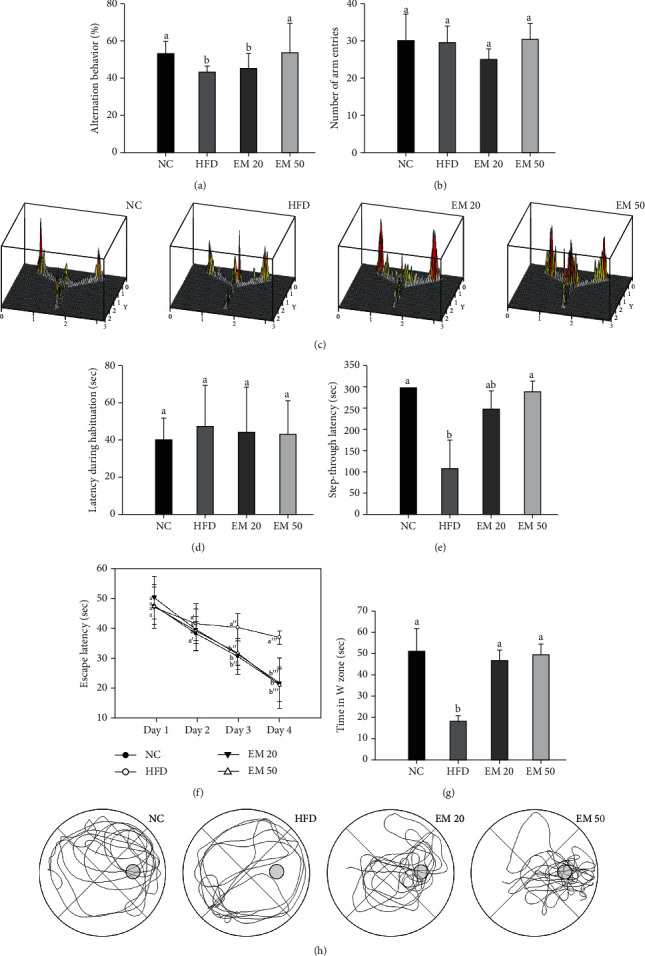
Effect of matcha on alternation behavior (a), number of arm entries (b), path tracing in Y-maze test (c), latency during habituation (d), step-through latency in passive avoidance (e), escape latency (f), time in W zone (g), and path tracing in the probe trial (h). The result shown are means ± SD (*n* = 7). Data were statistically considered at *P* < 0.05, and different small letters represent statistical differences.

Spatial learning and memory function were estimated using the Y-maze test (Figures 2(a)–2(c). There were no significant differences in the number of arm entries between all the groups (Figure 2(a)). The alternation behaviors of the EM 20 and EM 50 groups (45.67% and 54.27%, respectively) increased more than the HFD group (45.44%) (Figure 2(b)). Also, as the concentration of EM increased, it was seen that spatial learning and memory function were considerably improved (Figure 2(c)).

#### 3.2.2. Passive Avoidance Test

Short-term learning and memory ability were measured using the passive avoidance test (Figures 2(d) and 2(e)). The first day of step-through latency showed no significant change (Figure 2(d)). On the other hand, the second day of step-through latencies of the EM groups (248.86 s and 290.57 s) increased more than those of the HFD group (107.56 s) (Figure 2(e)).

#### 3.2.3. MWM Test

Long-term learning and memory ability were investigated using an MWM test (Figures 2(f)–2(h)). In the hidden test trial on the last training day, the escape latency of the EM groups (21.57 s and 20.87 s) decreased more than that of the HFD group (36.71 s) (Figure 2(f)). In the retention time in the W zone, the EM groups (46.75% and 49.50%) showed an increased retention time compared to the HFD group (18.50%) (Figure 2(g)). Comparing tracked movements, long-term learning and memory ability were considerably improved in the EM groups (Figure 2(h)).

### 3.3. Organ Weight Change

Weight changes of organs are presented in Table 1. The weights of the brain, testis, and pancreas showed no significant differences between all groups. However, the weight of the kidney (0.38 and 0.36 g), liver (1.92 and 1.32 g), and spleen (0.06 and 0.08 g) in the EM 20 and EM 50 groups was reduced compared to the HFD group (kidney, 0.40 g; liver, 2.21 g; spleen, 0.12 g). To estimate fat accumulation, perirenal, retroperitoneal, epididymal, and mesenteric fat mass were measured. The accumulation of perirenal (0.31 and 0.19 g), retroperitoneal (0.87 and 0.87 g), epididymal (1.79 and 1.46 g), and mesenteric (1.11 and 1.01 g) fat was suppressed compared to the HFD group (perirenal fat 0.40 g; retroperitoneal fat 2.11 g; epididymal fat 1.78 g; mesenteric fat 1.17 g).

### 3.4. Serum Biochemical

Serum biochemical indicators are shown in Table 2. The levels of GOT (87.60 and 69.00 U/L), GPT (72.00 and 45.60 U/L), and LDH (533.20 and 431.50 mg/dL) in the EM 20 and EM 50 groups were reduced compared to the HFD group (GOT, 126.20 U/L; GPT, 130.40 U/L; LDH, 649.60 mg/dL). The levels of TCHO (211.60 and 212.40 mg/dL), TG (132.20 and 131.20 mg/dL), and LDLC (23.88 and 23.24 mg/dL) in the EM 20 and EM 50 groups were reduced compared to the HFD group (THCO, 251.40 mg/dL; TG, 134.60 mg/dL; LDLC, 37.36 mg/dL). HTR (75.27 and 89.45%) in the EM 20 and EM 50 groups was improved compared to the HFD group (57.93%).

### 3.5. Protein Expression in White Adipose Tissue (WAT)

**Figure 3:**
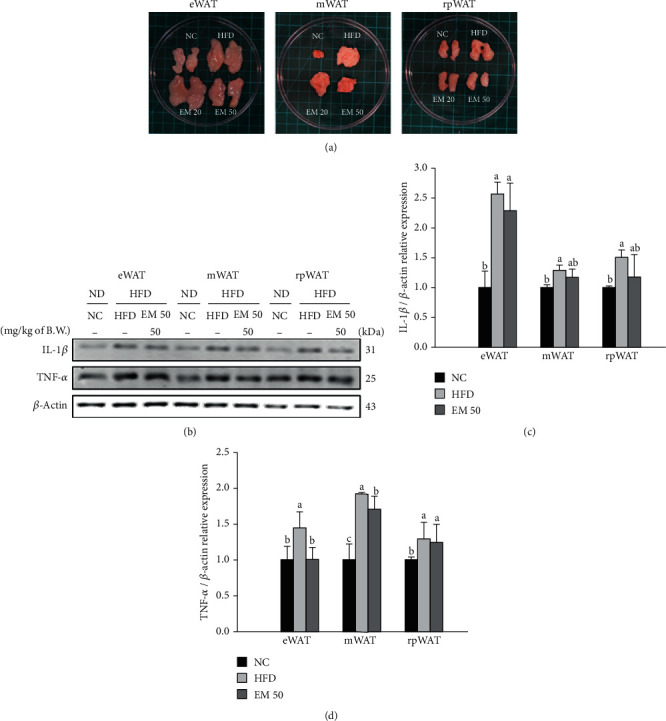
Effect of matcha on representative image of white adipose tissue (WAT) (a), protein expression of IL-1*β* and TNF-*α* (b), protein expression level of IL-1*β* (c), and TNF-*α* (d). The result shown are means ± SD (*n* = 3). Data were statistically considered at *P* < 0.05. Different small letters represent statistical differences. prWAT: perirenal white adipose tissue; rpWAT: retroperitoneal white adipose tissue; eWAT: epididymal white adipose tissue; mWAT: mesenteric white adipose tissue.

The eWAT, mWAT, and rpWAT mass of the HFD group increased higher than that of the NC group (Figure 3(a)). The EM groups statistically decreased those WAT masses. The IL-1*β* and TNF-*α* expression levels of eWAT, mWAT, and rpWAT are shown in Figure 3(b). The IL-1*β* expression levels of eWAT, mWAT, and rpWAT in the HFD group were significantly upregulated by 256.63%, 128.66%, and 150.98% compared to those in the NC group, respectively (Figure 3(c)). The EM 50 group statistically downregulated IL-1*β* expression levels by 10.49%, 9.19%, and 22.46% compared to those in the NC group, respectively. The TNF-*α* expression levels of eWAT, mWAT, and rpWAT in the HFD group were significantly upregulated by 143.90%, 192.72%, and 128.96% compared to those in the NC group, respectively (Figure 3(d)). The EM 50 group statistically downregulated TNF-*α* expression levels by 29.97%, 11.64%, and 3.34%, respectively.

### 3.6. Antioxidant System

#### 3.6.1. SOD Contents

**Figure 4:**
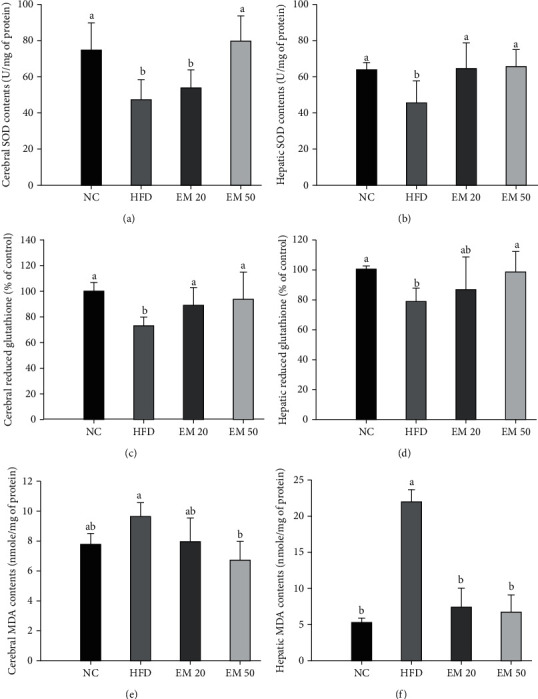
Effect of matcha on superoxide dismutase (SOD) content (a, b), reduced glutathione (GSH) content (c, d), and malondialdehyde (MDA) content (e, f) in brain and liver tissue. Results shown are mean ± SD (*n* = 7). Data were statistically considered at *P* < 0.05, and different small letters represent statistical differences.

Hepatic and cerebral SOD contents are presented in Figures 4(a) and 4(b). SOD contents of the EM 20 (64.81 and 54.41 U/mg of protein) and EDM 50 (65.60 and 79.86 U/mg of protein) groups increased compared to the HFD group (45.22 and 47.75 U/mg of protein) in liver and brain tissues, respectively.

#### 3.6.2. Reduced GSH

Reduced GSH contents are shown in Figures 4(c) and 4(d). The EM 20 (86.52 and 89.60% of control) and EM 50 (98.45 and 94.22% of control) groups showed a decrease of reduced GSH compared to the HFD group (78.61 and 73.45% of control) in liver and brain tissues, respectively.

#### 3.6.3. MDA Contents

MDA contents in the brain and liver are shown in Figures 4(e) and 4(f). The MDA contents in the brain and liver of EM 20 (7.38 and 7.98 mmole/mg of protein) and EM 50 (6.63 and 6.72 mmole/mg of protein) groups decreased more than in the HFD group (22.07 and 9.58 mmole/mg of protein) in liver and brain tissues, respectively.

### 3.7. Cerebral Cholinergic System

#### 3.7.1. ACh Contents

**Figure 5:**
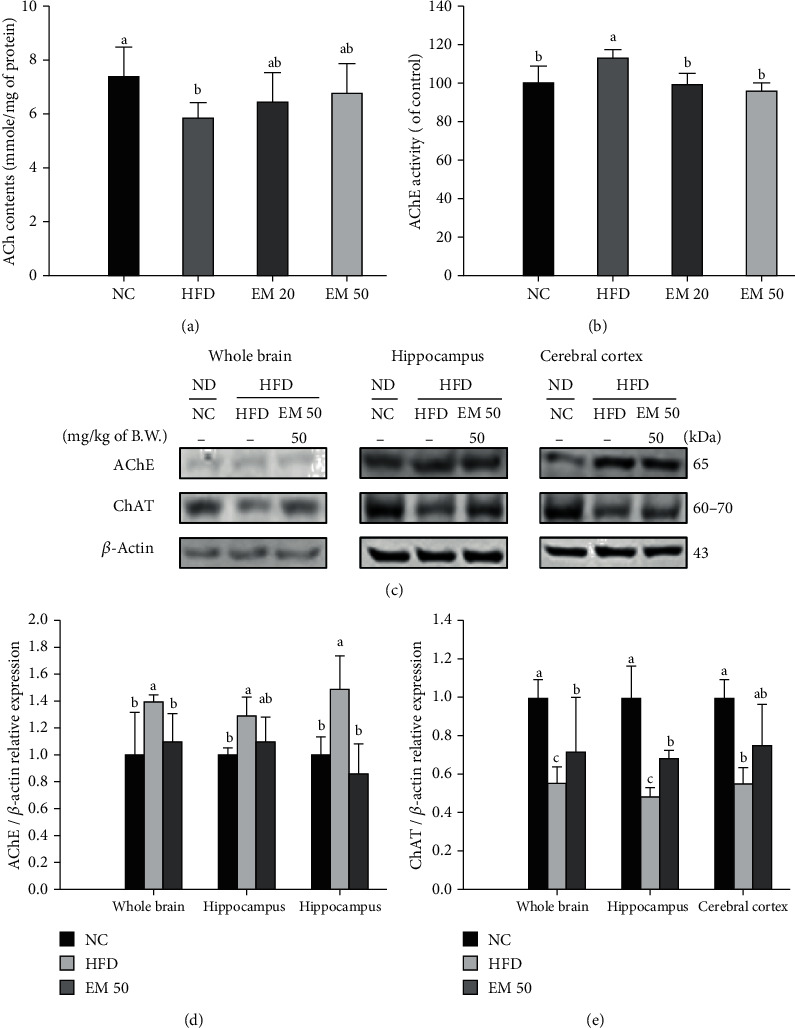
Effect of matcha on acetylcholine (ACh) contents (a), acetylcholinesterase (AChE) activities (b), protein expression of AChE and choline acyltransferase (ChAT) (c), protein expression level of AChE (d), and ChAT (e) in brain tissue. Results shown are mean ± SD ((a, b) 𝑛=7; (c–e) 𝑛=3). Data were statistically considered at *P* < 0.05, and different small letters represent statistical differences.

Cerebral ACh contents are shown in Figure 5(a). The EM 20 and EM 50 groups (6.47 and 6.76 mmole/mg of protein, respectively) showed a significant decrease of ACh contents compared to the HFD group (7.42 mmole/mg of protein).

#### 3.7.2. AChE Activities

AChE activities are shown in Figure 5(b). The AChE activities in the EM 20 and EM 50 groups (99.85% and 95.76%, respectively) were considerably lower than that of the HFD group (113.25%).

#### 3.7.3. Expression Levels of Cholinergic Enzyme

AChE and ChAT expression levels are shown in Figure 5(c). The AChE expression level in the HFD group was significantly upregulated by 139.56%, 129.27%, and 148.55% compared to that in the NC group in the whole brain, hippocampus, and cerebral cortex, respectively (Figure 5(d)). The EM 50 group statistically downregulated AChE expression levels by 21.19%, 20.86%, and 38.16%, respectively. The choline acyltransferase (ChAT) expression level in the HFD group was significantly reduced by 45.14%, 52.05%, and 44.90% compared to that in the NC group in the whole brain, hippocampus, and cerebral cortex, respectively (Figure 5(e)). The EM 50 group statistically upregulated ChAT expression levels by 71.82%, 67.83%, and 74.96%, respectively.

### 3.8. Mitochondrial Activity

#### 3.8.1. Mitochondrial ROS Contents

**Figure 6:**
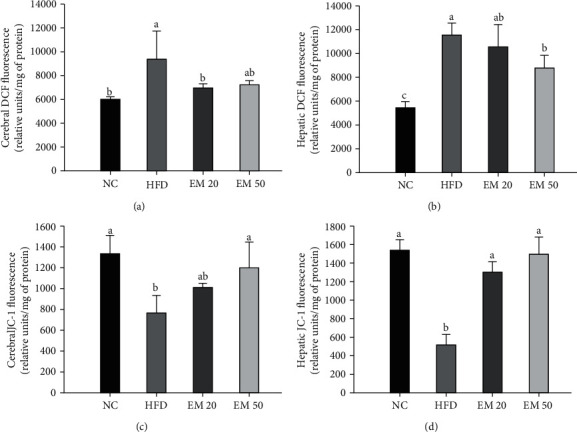
Effect of matcha on mitochondrial ROS production (a, b) and mitochondrial membrane potential (MMP) (c, d) in brain and liver tissue. Results shown are mean ± SD (*n* = 5). Data were statistically considered at *P* < 0.05, and different small letters represent statistical differences.

Mitochondrial ROS levels in the brain and liver are shown in Figures 6(a) and 6(b). The mitochondrial ROS content of the EM 20 (10564.42 and 7000.88 relative units/mg of protein) and EDM 50 (8848.16 and 7225.09 relative units/mg of protein) groups was reduced compared to the HFD group (11509.16 and 9381.66 relative units/mg of protein) in brain and liver tissues, respectively.

#### 3.8.2. MMP

MMP levels in the brain and liver are shown in Figures 6(c) and 6(d). The MMP level of the EM 20 (1303.12 and 1010.12 relative units/mg of protein) and EM 50 (1485.56 and 1190.68 relative units/mg of protein) groups increased compared to the HFD group (512.21 and 757.40 relative units/mg of protein) in brain and liver tissues, respectively.

### 3.9. Protein Expression in Hepatic Tissue

#### 3.9.1. Hepatic Inflammation

**Figure 7:**
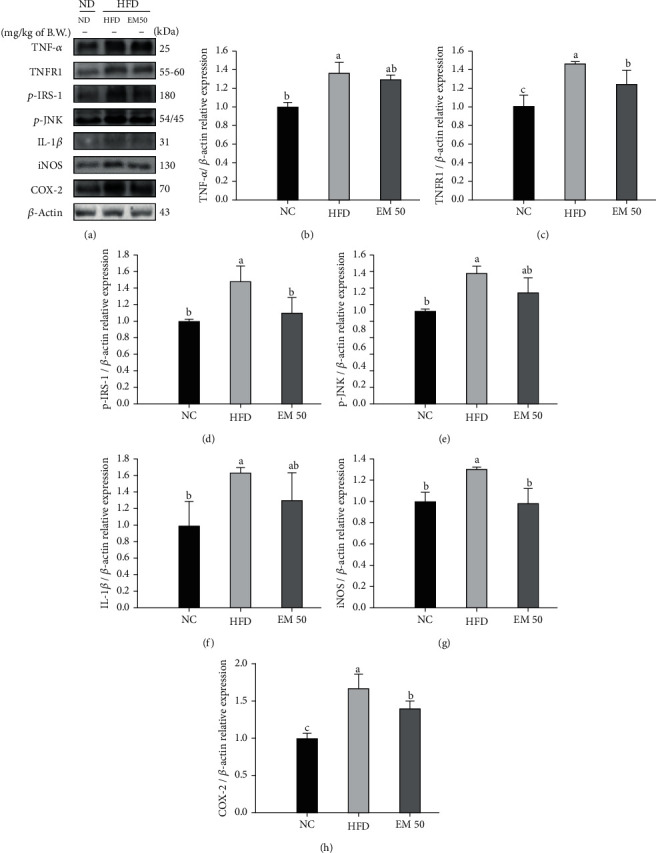
Hepatic protein expression levels of TNF-*α* (b), TNFR1 (c), *p*-IRS-1 (d), *p*-JNK (e), IL-1*β* (f), iNOS (g), and COX-2 (h). (a) Representative western blots for protein expression. Results shown are mean ± SD (*n* = 3). Data were statistically considered at *P* < 0.05, and different small letters represent statistical differences.

Hepatic protein expressions related to inflammation are presented in Figure 7. TNF-*α* (136.17%), tumor necrosis factor receptor 1 (TNFR1) (146.00%), phosphorylated insulin receptor substrate-1 (*p*-IRS-1) (148.26%), phospho-c-Jun N terminal kinase (*p*-JNK) (139.73%), IL-1*β* (162.72%), nitric oxide synthase (iNOS) (130.13%), and cyclooxygenase-2 (COX-2) (166.43%) expression levels in the HFD group were significantly upregulated compared to that in the NC group. The EM 50 group statistically downregulated TNF-*α* (4.50%), TNFR1 (14.86%), *p*-IRS-1 (26.43%), *p*-JNK (14.18%), IL-1*β* (20.14%), iNOS (24.74%), and COX-2 (15.53%) expression levels compared to the HFD group.

#### 3.9.2. Hepatic Protein Expressions Related to Lipid Synthesis and Cholesterol Metabolism

**Figure 8:**
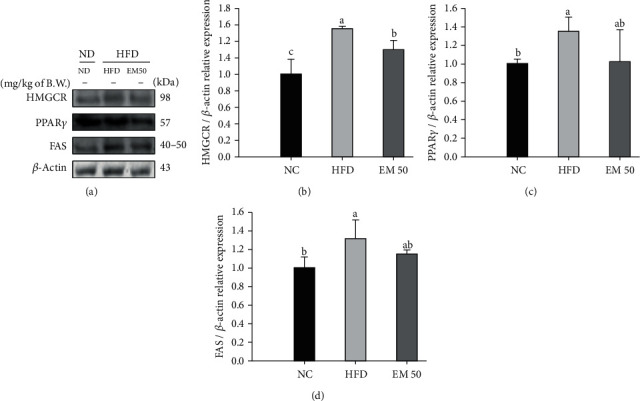
Hepatic protein expression levels of HMGCR (b), PPAR*γ* (c), and FAS (d). (a) Representative western blots for protein expression. Results shown are mean ± SD (*n* = 3). Data were statistically considered at *P* < 0.05, and different small letters represent statistical differences.

Hepatic protein expressions related to lipid synthesis and cholesterol metabolism are shown in Figure 8. HMG-CoA reductase (HMGCR) (154.56%), peroxisome proliferator-activated receptor-gamma (PPAR*γ*) (134.49%), and fatty acid synthase (FAS) (130.78%) expression levels in the HFD group were significantly upregulated compared to that in the NC group. The EM 50 group statistically downregulated HMGCR (16.45%), PPAR*γ* (23.93%), and FAS (12.46%) expression levels compared to the HFD group.

### 3.10. Protein Expression in Cerebral Tissue

#### 3.10.1. Tau Pathway

**Figure 9:**
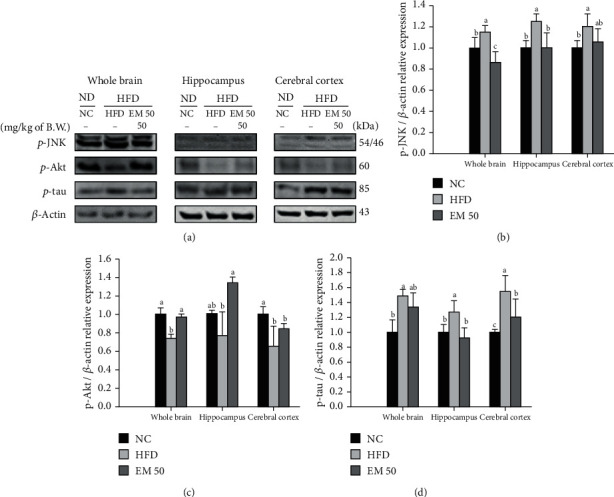
Cerebral protein expression levels of *p*-JNK (b), *p*-Akt (c), and *p*-tau (d). (a) Representative western blots for protein expression. Results shown are mean ± SD (*n* = 3). Data were statistically considered at *P* < 0.05, and different small letters represent statistical differences.

Cerebral protein expressions related to the tau pathway of the whole brain, hippocampus, and cerebral cortex are presented in Figure 9. *p*-JNK (115.22%, 124.96%, and 120.30%) and phosphorylated-tau (*p*-tau) (148.43%, 127.80%, and 155.42%) expression levels in the HFD group were significantly upregulated compared to that in the NC group. The EM 50 group statistically downregulated *p*-JNK (24.99%, 19.27%, and 11.98%) and *p*-tau (9.57%, 27.77%, and 22.33%) expression levels. Phosphoprotein kinase B (*p*-Akt) (26.37%, 23.46%, and 34.72%) expression levels in the HFD group were significantly reduced compared to that in the NC group. The EM 50 group statistically upregulated *p*-Akt (95.90%, 134.35%, and 84.44%) expression levels compared to the HFD group.

#### 3.10.2. Amyloid Beta Clearance Pathway

**Figure 10:**
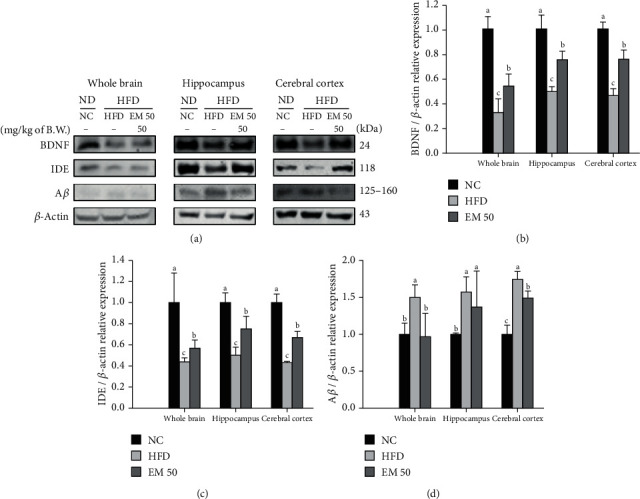
Cerebral protein expression levels of BDNF (b), IDE (c), and A*β* (d). (a) Representative western blots for protein expression. Results shown are mean ± SD (*n* = 3). Data were statistically considered at *P* < 0.05, and different small letters represent statistical differences.

Cerebral protein expressions related to amyloid-beta clearance of the whole brain, hippocampus, and cerebral cortex are presented in Figure 10. Cerebral protein expressions of the whole brain, hippocampus, and cerebral cortex are presented in Figure 10. Brain-derived neurotrophic factor (BDNF) (45.72%, 50.39%, and 53.26%) and IDE (55.85%, 43.36%, and 56.93%) expression levels in the HFD group were significantly reduced compared to that in the NC group. The EM 50 group statistically upregulated BDNF (54.28%, 75.28%, and 75.92%) and insulin-degrading enzyme (IDE) (56.73%, 74.95%, and 67.54%) expression levels. Amyloid beta (A*β*) (150.57%, 157.94%, and 174.90%) expression levels in the HFD group were significantly upregulated compared to that in the NC group. The EM 50 group statistically downregulated A*β* (35.93%, 13.28%, and 14.00%) expression levels compared to the HFD group.

#### 3.10.3. Neuroinflammation

**Figure 11:**
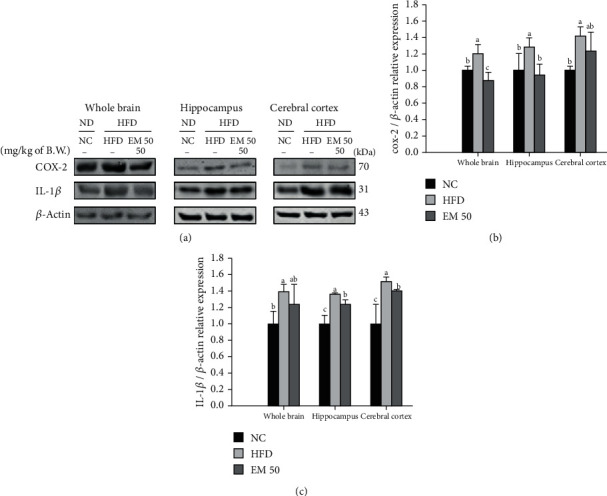
Cerebral protein expression levels of COX-2 (b) and IL-1*β* (c). (a) Representative western blots for protein expression. Results shown are mean ± SD (*n* = 3). Data were statistically considered at *P* < 0.05, and different small letters represent statistical differences.

Cerebral protein expressions related to neuroinflammation of the whole brain, hippocampus, and cerebral cortex are presented in Figure 11. COX-2 (119.55%, 128.45%, and 142.07%) and IL-1*β* (140.09%, 136.36%, and 151.79%) expression levels in the HFD group were significantly upregulated compared to that in the NC group. The EM 50 group statistically downregulated COX-2 (26.71%, 26.56%, and 13.27%) and IL-1*β* (11.59%, 8.86%, and 7.20%) expression levels compared to the HFD group.

## 4. Discussion

Diabetes is a chronic disease caused by excessive intake of HFD and can result in various metabolic diseases including insulin resistance, hyperglycemia, hyperlipidemia, and neurological dysfunction [4]. In particular, diabetes has been reported to cause an inflammatory reaction in the liver and insulin resistance in brain tissues, leading to cognitive impairment [8]. In particular, matcha used in this experiment is generally reported to exhibit considerable activity compared to other processed green teas [11]. In addition, it was confirmed that it presented significant antioxidant activity compared to leaf green tea through the preliminary study (Figure S1). Therefore, this study was conducted to evaluate the ameliorating effect of matcha on metabolic diseases derived from diabetes.

Long-term HFD intake is closely related to an increase in body weight. Excess carbohydrate intake promotes *de novo* lipogenesis, accumulating fat in the liver and adipocyte [29]. In addition, when insulin resistance is present in tissues, the blood insulin level also increases [4]. A high insulin level continuously promotes *de novo* lipogenesis, causing an increase in fat accumulation in liver and adipose tissue. The increase in the fat content of the liver and adipocytes eventually leads to an increase in body weight and glucose tolerance [29]. In addition, fat accumulation in the liver indicates the typical characteristics of diabetes such as hypercholesterolemia, hyperlipidemia, polyphagia, and hyperglycemia [4]. In diabetic status, insulin resistance is associated with increased activity of HMGCR in cholesterol synthesis [31]. Cholesterol synthesized by HMGCR under normal conditions constitutes a basic element of neurons, but excessive cholesterol production can lead to neuroinflammation and neuronal death [32]. Ultimately, HFD-induced glucose and lipid metabolism abnormalities are considered to be major mechanisms of damage to liver tissue and neurodegeneration [20]. On the other hand, intake of matcha showed suppressed glucose tolerance, reduction of fat mass, and an improvement effect on dyslipidemia. Catechins are reported to lower the content of TG and LDLC in serum and are reported to increase the content of HDLC to improve abnormal lipid metabolism [33]. In particular, EGCG inhibited the gene expressions of FAS, stearoyl-CoA desaturase-1 (SCD1), PPAR*γ*, and sterol regulatory element-binding protein 1 (SREBP1) involved in lipid metabolism and increased the excretion of free fatty acids [34]. The reduction of body weight and lipid improvement effect of matcha is considered to be due to the ameliorating effect of catechin contained in matcha.

HFD has been reported to cause disorders in spatial learning function [4]. HFD leads to the proinflammatory upregulating of the expression of cerebral inflammatory cytokines such as IL-1, interleukin-6 (IL-6), and TNF-*α* in microglial cells, astrocytes, and endothelial cells [35]. Also, cytokine receptors are located throughout the brain, but those are expressed in large amounts in the hippocampus, which are susceptible to damage by increased cytokines [36]. In addition, HFD induces inflammatory cytokines in the liver as well as brain tissue. Cytokines produced in the liver move through the blood to brain tissue, which cross the blood-brain barrier (BBB) and affect neuronal cells [6]. Increased cytokines cause oxidative stress, inflammatory reaction, and ultimately behavior and cognitive dysfunction. This inflammatory response particularly affects the hippocampus and cerebral cortex as components of memory formation and spatial information integration [35]. The brain tissue has a structure that is vulnerable to oxidative stress because it has higher content of unsaturated fatty acids than other tissues [7]. ROS and free radicals generated by HFD induce lipid peroxidation of cells, and those produce cytotoxic aldehyde products such as MDA and 4-hydroxylnonenal [35]. For this reason, HFD causes abnormal hippocampal synaptic plasticity and alterations in structure and function in normal brain tissue [37]. Cognitive impairment due to such oxidative damage can be improved through ingestion of a compound with antioxidant activity [38]. Matcha inhibits the dysfunction of the antioxidant system in the liver, brain, and blood and protects cognitive function [39]. In addition, EGCG contained in matcha improved the synaptic plasticity of the hippocampus *via* IRS/Akt and Erk/CREB/BDNF signaling pathways and reduced neuroinflammation [40, 41]. Similar to these results, consumption of matcha significantly improved HFD-induced cognitive impairment *via* protection of the antioxidant system. Based on these results, matcha statistically ameliorated HFD-induced cognitive impairment *via* improvement of plasticity in the hippocampus and cerebral cortex and antioxidant system in the liver and brain.

ACh and AChE in the central nervous system are strongly related to cognitive function. AChE, which divides ACh into acetate and choline, is normally located on the membrane of neuronal cells [42]. However, lipid peroxidation induced by HFD continuously increases the expression of AChE and promotes the breakdown of the neurotransmitter ACh [43]. In addition, HFD induces the aggregation of A*β*, inducing neuronal death in the brain. In particular, the association of A*β* and AChE occurs in the hippocampus, and this complex is known to be greater toxicity than normal A*β* [44]. Thus, HFD causes cognitive impairment through lipid peroxidation and aggregation of A*β*. However, green tea shows an improvement in cholinergic function, which inhibits AChE and butyrylcholinesterase (BuChE) and simultaneously inhibits *β*-secretase activity [45]. Also, green tea polyphenols are not only able to pass the BBB but also have physiological activity based on high antioxidant activity, which is considered to affect the protective effect of cholinergic systems [35]. To date, drugs used as A*β* inhibitors are synthetic chemicals that have a large molecular weight and are difficult to pass through the BBB, and various side effects have also been reported [45]. Therefore, similar to previous studies, matcha may be used as a natural material that can help cognitive function by improving the function of the cholinergic system.

Mitochondria are important organelles because they are a source of energy in cells. In mitochondria, ROS are produced during oxidative phosphorylation. However, excessive production of ROS leads to the excessive production of free fatty acid (FFA) [31]. This FFA continuously promotes ROS production and lipid peroxidation, causing damage to the antioxidant system. This mitochondrial damage leads to the release of cytochrome c and Ca^2+^ related to apoptosis and can result in the death of hepatocytes [46]. This suppresses the reduction of fat accumulation in liver tissue, and TG is continuously accumulated in the liver by reducing *β*-oxidation and ATP production [47]. Mitochondrial damage caused by HFD occurs in the brain as well as in the liver. Mitochondrial abnormalities lead to improper energy production and damaged calcium homeostasis [8]. The abnormal increase in intracellular Ca^2+^ induces the collapse of MMP and reduction of ATP production as well as the release of cytochrome c, which activates apoptosis of neurons [47]. To evaluate the ameliorating effect of matcha on mitochondrial deficit, ROS level and MMP in hepatic and cerebral mitochondria were assessed. Matcha improved hepatic mitochondria function, and it is considered to be able to suppress TG and fat accumulation. In addition, it may increase neuronal viability by protecting against cerebral mitochondrial damage and increasing the ATP production (Figure S2).

Ingestion of HFD indicates an increase in *Firmicutes* and a decrease in *Bacteroidetes* in the intestine and increases lipopolysaccharide (LPS), which activates a Toll-like receptor (TLR) signal pathway [48]. Absorbed LPS promotes the production of proinflammatory cytokines such as IL-1*β*, IL-6, and TNF-*α* in the liver [49]. Also, HFD increases the expression of TNFR1/2 to promote the absorption of TNF-*α*, which increases the expression of IL-1*β*, IL-6, and monocyte chemoattractant protein-1 (MCP-1) [50]. TNF-*α* significantly increases the FFA level in hepatocytes and significantly improves the expression of SREBP-1c, FAS, and HMGCR, promoting cholesterol synthesis and fatty acid synthesis [51]. This increase in fatty acid and cholesterol synthesis continuously causes insulin resistance, the activation of JNK, and the phosphorylation of IRS-1 [52]. Activated JNK activates the nuclear factor-*κ*B (NF-*κ*B) promoting the expression of iNOS and COX-2 to sustain the inflammatory response [48]. The inflammatory cytokines produced by those pathways move to the blood and easily pass through the BBB, causing an inflammatory response in neurons [35]. In particular, TNF-*α* and IL-1*β* increase the activity of GSK-3, promoting the phosphorylation of tau through the inhibition of Akt, and continuously increased *p*-tau exhibits neurotoxicity by aggregating neurofibrillary tangles [2]. These inflammatory reactions damage the function and structure of the hypothalamus, hippocampus, and cortex and induce CNS abnormalities such as cognitive dysfunction, behavioral abnormalities, and AD [37]. Also, in the hyperinsulinemia states, since the activity of IDE that degrades A*β* is inhibited, A*β* aggregation occurs in neurons [53]. In addition, HFD reduced BDNF, which is associated with hippocampal synaptic plasticity, leading to cognitive impairment [54]. On the other hand, green tea has been shown to inhibit the synthesis of fatty acid and de novo lipogenesis in HFD-induced mice through the activation of AMP-activated protein kinase (AMPK) [34]. Green tea polyphenol lowered the content of TNF-*α* in the liver and adipose tissues and improved hepatic steatosis by regulating the expression of SREBP-1c, FAS, and SCD1 [41]. The inhibition of inflammation indicates a decrease in cytokines released into the blood, which may lower the inflammatory response of brain tissue [49]. In addition, the consumption of green tea reduced the imbalanced production of cytokines such as TNF-*α*, IL-1*β*, and IL-6 in the cortex, cerebellum, and brainstem in obesity-induced mice [55]. Matcha lowered the content of cytokines in the liver and brain and effectively improved hyperinsulinemia by increasing the activity of the IDE. In addition, it is considered that matcha plays an important role in reducing *p*-tau and A*β* content.

## 5. Conclusions

In conclusion, this study confirmed that matcha can effectively protect against insulin resistance and cognitive impairment caused by hepatic and cerebral inflammatory responses in HFD-induced mice. In addition, it effectively ameliorated spatial cognitive function and short-term and long-term memory dysfunction by regulating the glucose tolerance and cholinergic system. The protective effect of the antioxidant system and mitochondrial function was confirmed by suppressing oxidative stress in liver and brain tissue. In addition, matcha improved inflammatory factors and insulin resistance in the liver and brain. Taken together, this study suggests that matcha could be used as a raw material for functional foods with antidiabetic activity and a protective effect on cognitive function.

## Figures and Tables

**Table 1:** Effect of matcha on changes of organ and white adipose tissue weight (g).

	NC	HFD	EM 20	EM 50
Brain	0.41 ± 0.05^a^	0.41 ± 0.02^a^	0.40 ± 0.03^a^	0.39 ± 0.03^a^
Kidney	0.33 ± 0.01^b^	0.40 ± 0.02^a^	0.38 ± 0.05^a^	0.36 ± 0.04^ab^
Testis	0.18 ± 0.02^a^	0.22 ± 0.07^a^	0.21 ± 0.03^a^	0.21 ± 0.04^a^
Liver	1.04 ± 0.02^b^	2.21 ± 0.32^a^	1.92 ± 0.43^a^	1.32 ± 0.34^b^
Spleen	0.07 ± 0.00^c^	0.12 ± 0.01^a^	0.09 ± 0.02^b^	0.08 ± 0.01^bc^
Pancreas	0.23 ± 0.02^a^	0.23 ± 0.02^a^	0.21 ± 0.03^a^	0.21 ± 0.03^a^
Perirenal fat	0.08 ± 0.04^b^	0.40 ± 0.10^a^	0.31 ± 0.06^a^	0.19 ± 0.13^b^
Retroperitoneal fat	0.25 ± 0.06^c^	2.11 ± 0.29^a^	0.87 ± 0.09^b^	0.87 ± 0.24^b^
Epididymal fat	1.24 ± 0.27^b^	1.78 ± 0.27^a^	1.79 ± 0.41^a^	1.46 ± 0.41^ab^
Mesenteric fat	0.35 ± 0.12^b^	1.17 ± 0.08^a^	1.11 ± 0.19^a^	1.01 ± 0.33^a^
Total fat	1.47 ± 0.25^c^	4.14 ± 0.18^a^	3.77 ± 0.60^ab^	3.44 ± 0.42^b^

Results shown are mean ± SD (*n* = 7). Data were statistically considered at *P* < 0.05, and different small letters represent statistical differences.

**Table 2:** Effect of matcha on serum biochemicals.

	NC	HFD	EM 20	EM 50
GOT (U/L)	43.80 ± 3.56^c^	126.20 ± 21.35^a^	87.60 ± 18.74^b^	69.00 ± 24.39^bc^
GPT (U/L)	28.80 ± 3.35^c^	130.40 ± 31.56^a^	72.00 ± 22.52^b^	49.60 ± 19.76^bc^
LDH (mg/dL)	154.00 ± 26.64^b^	649.60 ± 151.68^a^	533.20 ± 256.59^a^	431.50 ± 184.36^a^
TCHO (mg/dL)	117.20 ± 5.93^c^	251.40 ± 18.88^a^	211.60 ± 22.65^b^	212.40 ± 35.90^b^
TG (mg/dL)	122.40 ± 11.50^a^	134.60 ± 11.37^a^	132.20 ± 21.73^a^	131.20 ± 16.59^a^
HDLC (mg/dL)	76.60 ± 9.37^c^	161.80 ± 27.46^b^	196.00 ± 40.47^ab^	220.40 ± 36.06^a^
HTR (%)	75.08 ± 7.67^ab^	57.39 ± 11.89^b^	75.27 ± 3.43^ab^	89.45 ± 19.71^a^
LDLC (mg/dL)	16.88 ± 4.48^b^	37.36 ± 10.93^a^	23.88 ± 5.19^b^	23.24 ± 7.63^b^

Results shown are mean ± SD (*n* = 7). Data were statistically considered at *P* < 0.05, and different small letters represent statistical differences.

## Data Availability

The data used to support the findings of this study are available from the corresponding author upon request.

## Supplementary Materials

Supplementary Materials Table S1: list of primary antibodies and their information used in this study. Table S2: composition of experimental diets. Figure S1: antioxidant capacity of matcha. (a) Total phenolic content; (b) Total flavonoid content; (c) ABTS radical scavenging activity; (d) DPPH radical scavenging activity. Results shown are mean ± SD (*n* = 3). Data were statistically considered at *P* < 0.05. Figure S2: effect of matcha on mitochondrial ATP in brain. Results shown are mean ± SD (*n* = 5). Data were statistically considered at *P* < 0.05.
