# Supplementary material for: Matcha Improves Metabolic Imbalance-Induced Cognitive Dysfunction
Source: Oxid Med Cell Longev. 2020 Nov 28;2020:8882763. doi: 10.1155/2020/8882763 (PMC7719512; doi:10.1155/2020/8882763)
Supplement: Supplementary Materials — Table S1: list of primary antibodies and their information used in this study. Table S2: composition of experimental diets. Figure S1: antioxidant capacity of matcha. (a) Total phenolic content; (b) Total flavonoid content; (c) ABTS radical scavenging activity; (d) DPPH radical scavenging activity. Results shown are mean ± SD (n = 3). Data were statistically considered at P < 0.05. Figure S2: effect of matcha on mitochondrial ATP in brain. Results shown are mean ± SD (n = 5). Data were statistically considered at P < 0.05. [file 8882763.f1.docx]

Table S1: List of primary antibodies and their information used in this study

| **Antibody** | **Catalog** | **Conc.** | **Manufacturer** |
| --- | --- | --- | --- |
| β‐actin | sc‐69879 | 1:1000 | Santa Cruz Biotech. (Dallas, TX, USA) |
| AChE | sc-373901 | 1:1000 | Santa Cruz Biotech. (Dallas, TX, USA) |
| p-JNK | sc‐6254 | 1:1000 | Santa Cruz Biotech. (Dallas, TX, USA) |
| p-Akt | sc-514032 | 1:1000 | Santa Cruz Biotech. (Dallas, TX, USA) |
| p-tau | sc‐12952 | 1:1000 | Santa Cruz Biotech. (Dallas, TX, USA) |
| Aβ | sc-28365 | 1:1000 | Santa Cruz Biotech. (Dallas, TX, USA) |
| p-IRS-1 | sc-33956 | 1:1000 | Santa Cruz Biotech. (Dallas, TX, USA) |
| IDE | sc-393887 | 1:1000 | Santa Cruz Biotech. (Dallas, TX, USA) |
| COX-2 | sc-376861 | 1:1000 | Santa Cruz Biotech. (Dallas, TX, USA) |
| iNOS | sc-7271 | 1:1000 | Santa Cruz Biotech. (Dallas, TX, USA) |
| IL-1β | sc-4592 | 1:1000 | Santa Cruz Biotech. (Dallas, TX, USA) |
| ChAT | 20747-1AP | 1:1000 | Bioneer (Daejeon, Korea) |
| TNF-α | 5178SC | 1:1000 | Cell Signaling Tech. (Danvers, MA, USA) |
| FAS | NB400-114 | 1:1000 | Novus Biologicals (Centennial, CO, USA) |
| PPARγ | NBP2-22106 | 1:1000 | Novus Biologicals (Centennial, CO, USA) |
| p-GSK-3β | CSB-RA009963A09phHU | 1:1000 | Cusabio (Hubei, China) |
| Caspase-3 | CSB-PA05689A0Rb | 1:1000 | Cusabio (Hubei, China) |
| BDNF | CSB-PA05775A0Rb | 1:1000 | Cusabio (Hubei, China) |
| TNFR1 | CSB-PA621879EA01HU | 1:1000 | Cusabio (Hubei, China) |
| HMGCR | CSB-PA010565LA01HU | 1:1000 | Cusabio (Hubei, China) |

Table S2: Composition of experimental diets

| **Diet** | **Normal diet (D12450B)** | | **High-fat diet (D12492)** | |
| --- | --- | --- | --- | --- |
|  | gram% | kcal% | gram% | kcal% |
| Protein | 19.2 | 20 | 26.2 | 20 |
| Carbohydrate | 67.3 | 70 | 26.3 | 20 |
| Fat | 4.3 | 10 | 34.9 | 60 |
|  | 3.85 (kcal/g) | | 5.24 (kcal/g) | |
| Ingredient | gram | kcal | gram | kcal |
| Casein | 200 | 800 | 200 | 800 |
| L-Cystine | 3 | 12 | 3 | 12 |
| Corn Starch | 315 | 1,260 | 0 | 0 |
| Maltodextrin 10 | 35 | 140 | 125 | 500 |
| Sucrose | 350 | 1,400 | 68.8 | 275.2 |
| Cellulose | 50 | 0 | 50 | 0 |
| Soybean Oil | 25 | 225 | 25 | 225 |
| Lard | 20 | 180 | 245 | 2205 |
| Mineral Mix | 10 | 0 | 10 | 0 |
| DiCalcium Phosphate | 13 | 0 | 13 | 0 |
| Calcium Carbonate | 5.5 | 0 | 5.5 | 0 |
| Potassium Citrate | 16.5 | 0 | 16.5 | 0 |
| Vtamin Mix | 10 | 40 | 10 | 40 |
| Choline Bitartate | 2 | 0 | 2 | 0 |

*Animal diets were purchased from Research Diets INC (New Brunswick, NJ, USA).

**
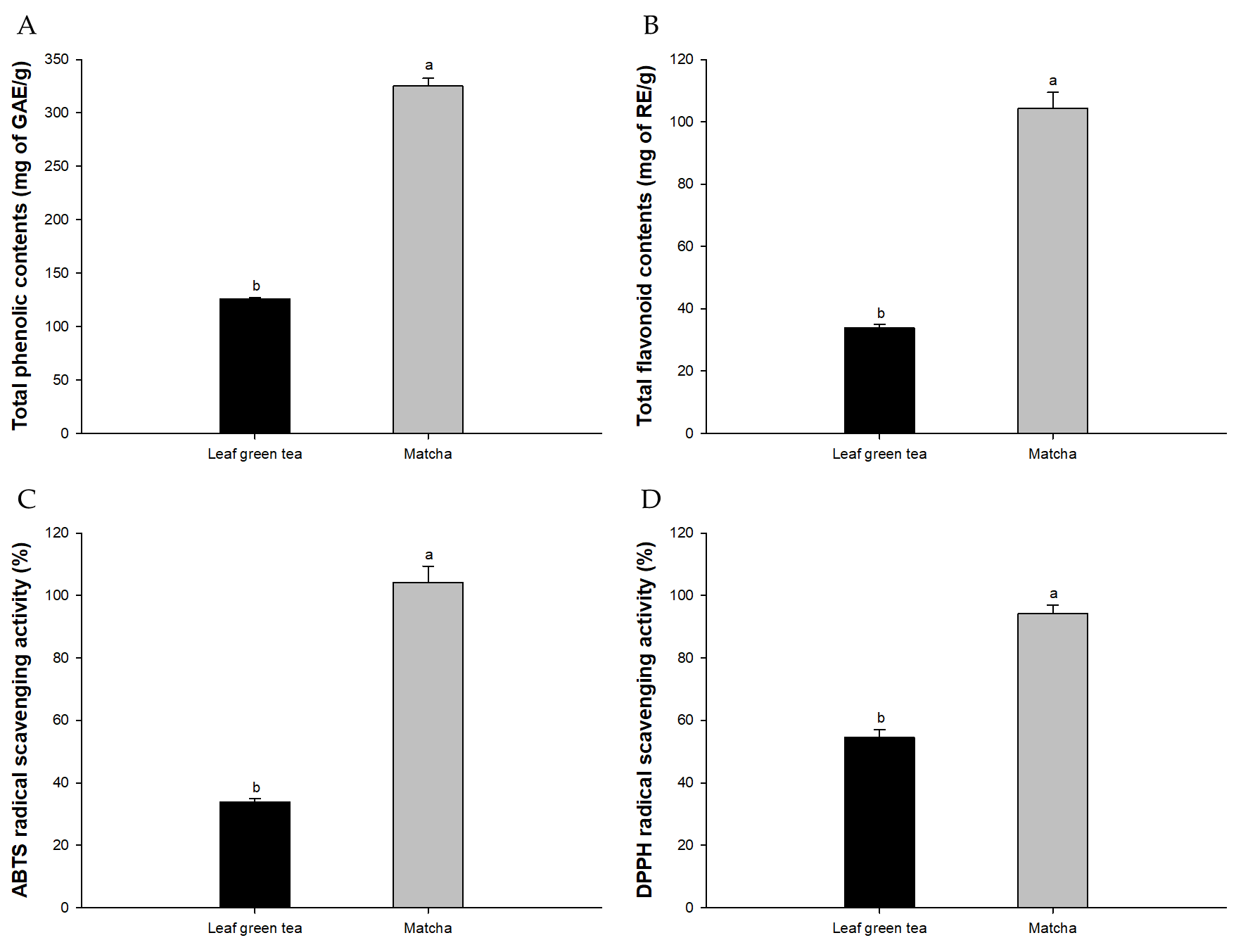
**

Figure S1: Antioxidant capacity of matcha. A, total phenolic content; B, total flavonoid content; C, ABTS radical scavenging activity; D, DPPH radical scavenging activity. Results shown are mean±SD (𝑛=3). Data were statistically considered at *P* < 0.05.

Figure S2: Effect of matcha on mitochondrial ATP in brain. Results shown are mean±SD (𝑛=5). Data were statistically considered at *P* < 0.05.
